# Influenza Vaccination and the Risk of Ventricular Arrhythmias in Patients With Chronic Obstructive Pulmonary Disease: A Population-Based Longitudinal Study

**DOI:** 10.3389/fcvm.2021.731844

**Published:** 2021-10-14

**Authors:** Chun-Chao Chen, Cheng-Hsin Lin, Wen-Rui Hao, Jong-Shiuan Yeh, Kuang-Hsing Chiang, Yu-Ann Fang, Chun-Chih Chiu, Tsung Yeh Yang, Yu-Wei Wu, Ju-Chi Liu

**Affiliations:** ^1^Division of Cardiology, Department of Internal Medicine, Shuang Ho Hospital, Taipei Medical University, New Taipei City, Taiwan; ^2^Taipei Heart Institute, Taipei Medical University, Taipei, Taiwan; ^3^Division of Cardiology, Department of Internal Medicine, School of Medicine, College of Medicine, Taipei Medical University, Taipei, Taiwan; ^4^Division of Cardiovascular Surgery, Department of Surgery, Shuang Ho Hospital, Taipei Medical University, New Taipei City, Taiwan; ^5^Division of Cardiovascular Surgery, Department of Surgery, School of Medicine, College of Medicine, Taipei Medical University, Taipei, Taiwan; ^6^College of Medicine, Graduate Institute of Clinical Medicine, Taipei Medical University, Taipei, Taiwan; ^7^Division of Cardiovascular Medicine, Department of Internal Medicine, Taipei Municipal Wan-Fang Hospital, Taipei, Taiwan; ^8^Division of Cardiology, Department of Internal Medicine, Taipei Medical University Hospital, Taipei, Taiwan; ^9^Graduate Institute of Biomedical Electronics and Bioinformatics, National Taiwan University, Taipei, Taiwan; ^10^College of Medical Science and Technology, Graduate Institute of Biomedical Informatics, Taipei Medical University, Taipei, Taiwan; ^11^Clinical Big Data Research Center, Taipei Medical University Hospital, Taipei, Taiwan

**Keywords:** chronic obstructive pulmonary disease, influenza vaccination, ventricular arrhythmia, ventricular fibrillation, ventricular tachycardia

## Abstract

**Backgrounds:** Influenza vaccination could decrease the risk of major cardiac events in patients with chronic obstructive pulmonary disease (COPD). However, the effects of the vaccine on decreasing the risk of ventricular arrhythmia (VA) development in such patients remain unclear.

**Methods:** We retrospectively analyzed the data of 18,658 patients with COPD (≥55 years old) from the National Health Insurance Research Database from January 1, 2001, to December 31, 2012. After a 1:1 propensity score matching by the year of diagnosis, we divided the patients into vaccinated and unvaccinated groups. Time-varying Cox proportional hazards regression was applied to assess the time to event hazards of influenza vaccination exposure.

**Results:** The risk of VA occurrence was significantly lower in the vaccinated group during influenza season and all seasons [adjusted hazard ratio (aHR): 0.62, 95% CI: 0.41–0.95; aHR: 0.69, 95% CI: 0.44–1.08; and aHR: 0.65, 95% CI: 0.48–0.89, in the influenza season, non-influenza season, and all seasons, respectively]. Among patients with CHA_2_DS_2_-VASc scores (conditions and characteristics included congestive heart failure, hypertension, diabetes, stroke, vascular disease, age, and sex) of 2–3, receiving one time and two to three times of influenza vaccination were associated with lower risk of VA occurrence in all seasons (aHR: 0.28, 95% CI: 0.10–0.80; aHR: 0.27, 95% CI: 0.10–0.68, respectively). Among patients without stroke, peripheral vascular disease, and diabetes, a lower risk of VA occurrence after receiving one and two to three times vaccination was observed in all seasons. Among patients with a history of asthma and patients without a history of heart failure, ischemic heart disease, angina hypertension, or renal failure, a significantly lower risk of VA occurrence was observed after the first time of vaccination in all seasons.

**Conclusions:** Influenza vaccination may be associated with lower risks of VA among patients with COPD aged 55–74. Further investigation is still needed to resolve this clinical question.

## Introduction

Chronic obstructive pulmonary disease (COPD) is a worldwide health burden and has risen from the sixth to the third most common cause of death in the past 30 years (1). Studies have revealed that cardiovascular disease is a major cause of death among patients with COPD (2). Because patients with COPD often have similar comorbidities that increase the risk of developing cardiovascular disease, the risks of heart failure, myocardial infarction, and arrhythmia also increase (3, 4). Although supraventricular arrhythmia is the most common form of arrhythmia in patients with COPD arrhythmia (5), the risk of ventricular arrhythmia (VA) is also high (6, 7). Moreover, the risk of sudden death is significantly heightened in patients with COPD (5, 8). In the acute exacerbation of COPD, the most common type of cardiac arrhythmia is premature ventricular contractions (9). Increased QT dispersion, autonomic function, hypoxemia, and hypercapnia were the proposed mechanisms of vulnerability to VA in patients with COPD (7, 10, 11). VA is also a possible risk factor of mortality in patients with COPD during acute exacerbation (12).

Influenza is a common and severe viral infection among patients with COPD. It increases the risk of not only acute respiratory failure but also acute cardiovascular events (13). The risk of myocardial infarction and sudden cardiac death decreases upon influenza vaccination (13–15). However, the association of influenza vaccination and VA among patients with COPD is still unclear. Therefore, our study investigated the association between influenza vaccination and the occurrence of VA in a real-world setting.

## Methods

The National Health Insurance (NHI) program established in 1995 provides health insurance coverage for more than 98% of the Taiwanese population (more than 23 million people). The NHI Research Database (NHIRD) is maintained by the Health and Welfare Data Science Center and has been extensively analyzed and validated by earlier studies (16). In Taiwan, the influenza vaccination is provided free of charge for adults aged over 50 years with high-risk comorbidities (i.e., diabetes, chronic liver disease, cirrhosis, cardiovascular disease, or chronic pulmonary disease). The NHIRD research committee and the Joint Institutional Review Board of Taipei Medical University approved our study protocol (TMU-JIRB No. N201905004).

### Study Cohort and Study Design

**Figure 1:**
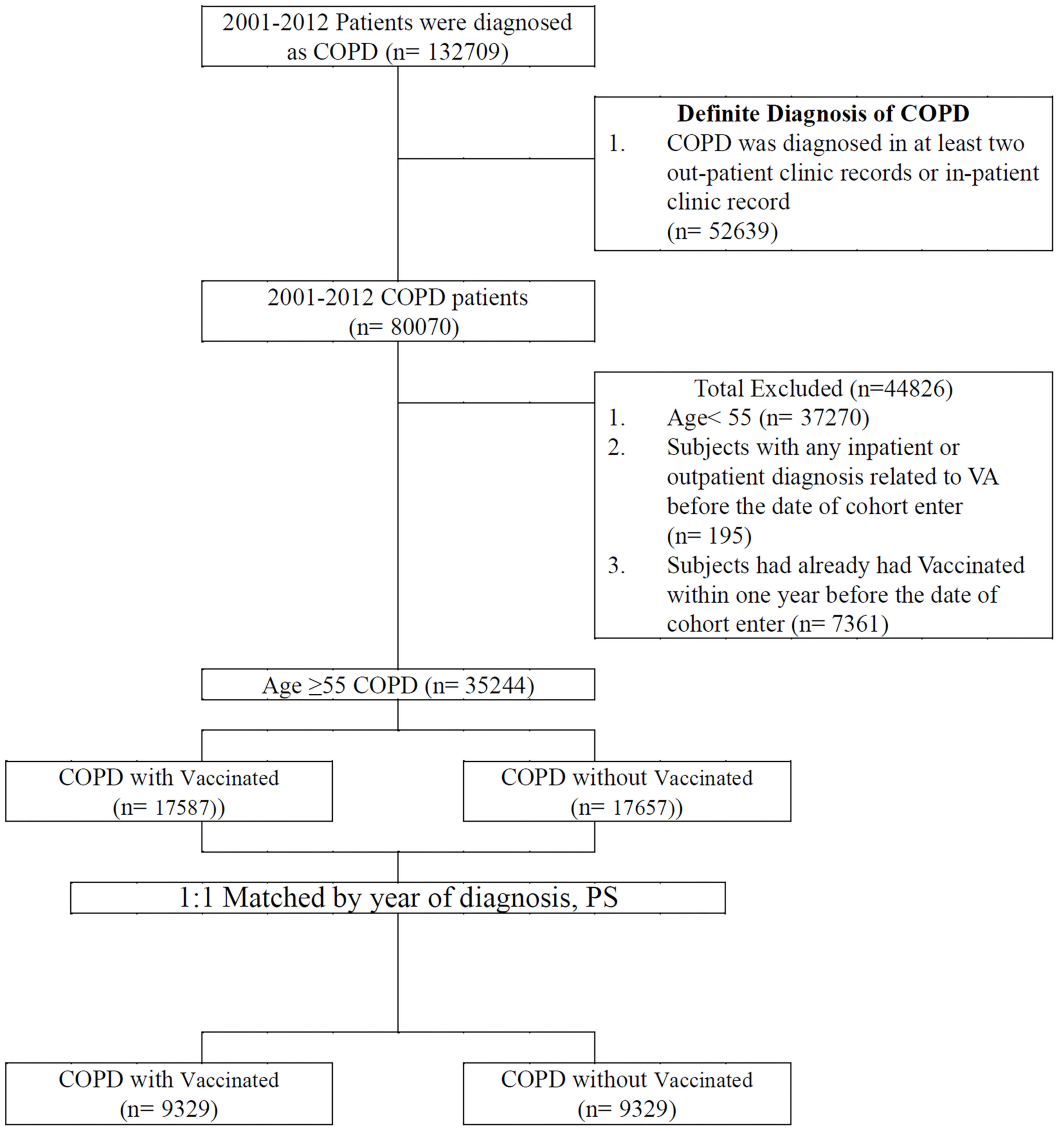
Data selection process.

The patients included in the study were diagnosed as having COPD between January 1, 2001, and December 31, 2012. The positive predictive value of the ICD-9-CM codes in COPD diagnosis was previously validated (17). In addition, they had at least two COPD diagnoses as inpatients or outpatients (18) and were aged ≥55 years. Vaccination status was identified by code V048 and/or the use of the vaccine (confirmed by drug codes). A comparison cohort was selected by blinding the outcome using a SAS (Statistical Analysis Software) software package. Each patient with COPD who received vaccination underwent propensity score–based matching for sociodemographic characteristics (age, gender, urbanization level, monthly income); comorbidities (asthma [ICD-9-CM code 493], heart failure [ICD-9-CM code 428], acute myocardial infarction [ICD-9-CM code 410], stroke [ICD-9-CM code 430-438], ischemic heart disease [ICD-9-CM code 414], angina [ICD-9-CM code 413], peripheral vascular disease [ICD-9-CM code 448], hypertension [ICD-9-CM code 405], diabetes [ICD-9-CM code 250], depression [ICD-9-CM code 296.2; 303.4; 296.3; 311], renal failure [ICD-9-CM code 403, 404, 582, 583, 585, 586, 588], chronic liver disease [ICD-9-CM code 456, 571, 572], dementia [ICD-9-CM code 290, 797]); CHA_2_DS_2_-VASc score; COPD severity (defined as COPD-related inpatient visits) (19); COPD medications (short-acting beta agonist [SABA], long-acting beta-agonist [LABA], long-acting muscarinic antagonist [LAMA], inhaled corticosteroids [ICS]); and total numbers of COPD medications with those who did not receive vaccinations for comparing VA incidence. After a 1:1 propensity score matching by the year of diagnosis, we divided the patients into vaccinated and unvaccinated groups. Any individual with prior VA in a matched pair was excluded. The cohort entry date (index date) for patients receiving vaccines was defined as the vaccine initiation date. In the matched pairs, the participants who received vaccines and those who did not receive vaccines were given the same index date (vaccine initiation date) for follow-up. All patients were followed up until one of the following occurred: an initial diagnosis of ventricular tachycardia (ICD-9-CM code 427.1), ventricular fibrillation, or ventricular flutter (ICD-9-CM code 427.4, 427.41, or 427.42), loss to follow-up, death, withdrawal from the NHIRD or December 31, 2012 (Figure 1).

### Potential Confounders

The potential confounders of our cohort were based on sociodemographic characteristics (age, gender, urbanization level, and monthly income), comorbidities (asthma, heart failure, acute myocardial infarction, stroke, ischemic heart disease, angina, peripheral vascular disease, hypertension, diabetes, depression, renal failure, chronic liver disease, dementia, and CHA_2_DS_2_-VASc score), COPD severity and treatment (COPD-related inpatient visits, COPD medications, total number of COPD medications), and medication use (aspirin, statin, renin-angiotensin-aldosterone system inhibitor [RAASI], and Class I [sodium channel blocker], Class II [beta blocker], Class III [potassium channel blocker], and Class IV [calcium channel blocker] antiarrhythmic agents).

### Statistical Analysis

All baseline patient characteristics mentioned earlier are described in Table 1. Categorical variables are reported as numbers and percentages, and quantitative variables are presented as mean ± SDs. The balance of characteristics was assessed by estimating standardized differences (StDiffs) between the vaccinated group and the unvaccinated group. A time-varying Cox proportional hazard model was used to calculate the hazard ratios (HRs) to determine the risk of VA between the vaccinated and unvaccinated groups (20). The aforementioned confounders were treated as a fixed effect in the Cox model, while influenza vaccination exposure in the follow-up period was the random effect. In the sensitivity analyses, external adjustments clarified the effects of covariates in epidemiological database studies. Thus, in the sensitivity analysis, patients were stratified to estimate the association of age, gender, CHA2DS2-VASc score, COPD-related inpatient visits, asthma, heart failure, stroke, ischemic heart disease, angina, peripheral vascular disease, hypertension, diabetes, renal failure, and chronic liver disease with the incidence of VA in different models. The VA-free survival rate in the vaccinated and unvaccinated patients with COPD was calculated using the Kaplan–Meier method. Influenza season is defined as the period between October and March according to the Taiwan Centers for Disease Control. All analyses were performed using SAS (Version 9.4, SAS, Cary, NC, USA); a two-tailed *P*-value of < 0.05 was considered significant.

**Table 1:** Pooled baseline characteristics balance before and after propensity score matching.

	**After matching**				
	**COPD without vaccinated**		**COPD with vaccinated**		**Standardized difference**
	***n*** **=** **(9,329)**		***n*** **=** **(9,329)**		
	***n***	**%**	***n***	**%**	
**COPD-related inpatient visits**					
0	6,804	72.93	6,978	74.80	0.042
1	1,316	14.11	1,179	12.64	−0.043
≥2	1,09	12.96	1,172	12.56	−0.012
**Age, years (Mean** **±** **SD)**	69.82 ± 9.31		70.13 ± 8.98		0.033
55–64	3,459	37.08	3,368	36.10	−0.020
65–74	3,027	32.45	3,115	33.39	0.020
≥75	2,843	30.47	2,846	30.51	0.001
**Gender**					
Female	3,953	42.37	4,096	43.91	0.031
Male	5,376	57.63	5,233	56.09	−0.031
**CHA2DS2-VASc score**					
0	1,318	14.13	1,124	12.05	−0.062
1	1,721	18.45	1,572	16.85	−0.042
2–3	3,310	35.48	3,385	36.28	0.017
≥4	2,980	31.94	3,248	34.82	0.061
**Comorbidities**					
Asthma	3,970	42.56	4,400	47.16	0.093
Heart failure	1,087	11.65	1,136	12.18	0.016
Acute myocardial infarction	180	1.93	194	2.08	0.011
Stroke	2,300	24.65	2,559	27.43	0.063
Ischemic heart disease	3,015	32.32	3,391	36.35	0.085
Angina	1,003	10.75	1,203	12.90	0.066
Peripheral vascular disease	940	10.08	1,051	11.27	0.039
Hypertension	5,504	59.00	5,871	62.93	0.081
Diabetes	2,657	28.48	2,888	30.96	0.054
Depression	347	3.72	447	4.79	0.053
Renal failure	1,349	14.46	1,352	14.49	0.001
Chronic liver disease	2,367	25.37	2,634	28.23	0.065
Dementia	600	6.43	679	7.28	0.034
**Level of urbanization**					
Urban	6,495	69.62	6,166	66.09	−0.076
Suburban	1,969	21.11	2,015	21.60	0.012
Rural	865	9.27	1,148	12.31	0.098
**Monthly income (NT$)**					
0	1,155	12.38	981	10.52	−0.059
1–33,300	6,249	66.98	6,366	68.24	0.027
≥33,301	1,925	20.63	1,982	21.25	0.015
**COPD medications**					
SABA	2,261	24.24	2,336	25.04	0.019
SABA+SAMA	1,913	20.51	1,962	21.03	0.013
LABA (Ultra-LABA)	109	1.17	125	1.34	0.015
LAMA	457	4.90	577	6.19	0.056
ICS	955	10.24	1,007	10.79	0.018
ICS+LABA	1,273	13.65	1,429	15.32	0.048
**Total COPD medications**					
0	5,484	58.78	5,377	57.64	−0.023
1	2,014	21.59	1,974	21.16	−0.010
≥2	1,831	19.63	1,978	21.20	0.039
Medication use^*^					
Aspirin	3,103	33.26	3,853	41.30	0.167
Statin	2,006	21.50	2,640	28.30	0.158
RAASI	4,197	44.99	4,963	53.20	0.165
Class1	194	2.08	276	2.96	0.061
Class2	2,286	24.50	2,775	29.75	0.118
Class3	640	6.86	627	6.72	−0.006
Class4	1,451	15.55	1,692	18.14	0.069

*Standardized difference = difference in mean or proportions divided by the standard error; imbalance between groups was defined as an absolute value >0.10 (corresponding to a small effect size)*.

*Propensity score-matched is adjusted for COPD-related inpatient visits, COPD medications, total COPD medications, age, gender, CHA2DS2-VASc score, asthma, heart failure, acute myocardial infarction, stroke, ischemic heart disease, angina, peripheral vascular disease, hypertension, diabetes, depression, renal failure, chronic liver disease, dementia, level of urbanization, monthly income*.

* *Propensity score matching did not adjust these variables*.

*COPD, chronic obstructive pulmonary disease; SABA, short-acting beta agonist; SAMA, short-acting muscarinic antagonist; LABA, long-acting beta-agonist; LAMA, long-acting muscarinic antagonist; ICS, inhaled corticosteroids; RAASI, renin-angiotensin-aldosterone system inhibitor*.

## Results

In total, 18,658 patients were enrolled and matched pairs were obtained comprising individuals in the vaccinated and comparison cohorts. Table 1 shows the baseline characteristics of both groups after propensity score matching.

### Association Between Influenza Vaccination and VA on Different Age Groups and Genders

**Figure 2:**
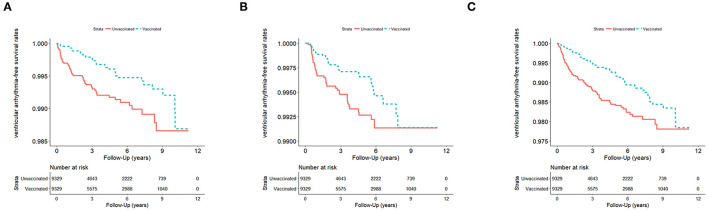
**(A)** VA events in influenza season (*n* = 18,658; January 1, 2001–December 31, 2012) in Taiwan, stratified by vaccinated and unvaccinated groups (log rank test, χ^2^ = 10.686; df = 1; *p* = 0.001). **(B)** VA events in non-influenza season (*n* = 18,658) from January 1, 2001 to December 31, 2012, in Taiwan, stratified by vaccinated and unvaccinated groups (log rank test, χ^2^ = 4.101; df = 1; *p* = 0.063). **(C)** VA events in all seasons (*n* = 18,658) from January 1, 2001 to December 31, 2012, in Taiwan, stratified by vaccinated and unvaccinated groups (log rank test, χ^2^ = 14.272; df = 1; *p* < 0.001). VA, ventricular arrhythmia.

Table 2 demonstrates the risk of VA between vaccination and non-vaccination groups stratified by age and gender. We observed a significantly low occurrence of VA during influenza season and all seasons (aHR: 0.62 [95% CI: 0.41–0.95], 0.69 [95% CI: 0.44–1.08], 0.65 [95% CI: 0.48–0.89], in influenza season, non-influenza season, and all seasons, respectively) (Figure 2). In patients with COPD between 55 and 64 years old, a significant lower risk of VA occurrence was observed after receiving vaccination in non-influenza season and all seasons (aHR: 0.22 [95% CI: 0.07–0.68], 0.33 [95% CI: 0.16–0.69], respectively). In patients with ages between 65 and 74 years, a lower risk of VA occurrence in the vaccinated group was observed in all seasons. In patients with age more than 75 years, there was no significant difference in VA occurrence after vaccination in the influenza season, non-influenza season, and all seasons.

**Table 2:** Risk of ventricular arrhythmia among unvaccinated and vaccinated in study cohort.

**All group (*n* = 18,658)**	**Unvaccinated (Total follow-up 40796.4 person-years)**		**Vaccinated (Total follow-up 39432.1 person-years)**		**Unadjusted HR (95% C.I.)**	**Adjusted HR^†^ (95% C.I.)**
	**No. of patients with VA**	**Incidence rate (per 10^**5**^ person-years) (95% C.I.)**	**No. of patients with VA**	**Incidence rate (per 10^**5**^ person-years) (95% C.I.)**		
**Whole cohort**						
Influenza season	60	147.1 (109.9, 184.3)	35	88.8 (59.4, 118.2)	0.59 (0.39, 0.90)^*^	0.62 (0.41, 0.95)^*^
Non-influenza season	46	112.8 (80.2, 145.3)	34	86.2 (57.2, 115.2)	0.71 (0.46, 1.11)	0.69 (0.44, 1.08)
All season	106	259.8 (210.4, 309.3)	69	175.0 (133.7, 216.3)	0.64 (0.47, 0.87)^**^	0.65 (0.48, 0.89)^**^
**Age, 55–64** ^a^						
Influenza season	15	98.5 (48.6, 148.3)	6	44.7 (8.9, 80.4)	0.42 (0.16, 1.08)	0.48 (0.18, 1.29)
Non-influenza season	17	111.6 (58.5, 164.6)	4	29.8 (0.6, 58.9)	0.26 (0.09, 0.78)^*^	0.22 (0.07, 0.68)^**^
All season	32	210.0 (137.3, 282.8)	10	74.4 (28.3, 120.6)	0.34 (0.17, 0.69)^**^	0.33 (0.16, 0.69)^**^
**Age, 65–74** ^b^						
Influenza season	25	166.4 (101.1, 231.6)	12	79.8 (34.7, 125.0)	0.48 (0.24, 0.97)^*^	0.51 (0.25, 1.03)
Non-influenza season	20	133.1 (74.8, 191.4)	11	73.2 (29.9, 116.4)	0.52 (0.25, 1.08)	0.48 (0.22, 1.02)
All season	45	299.5 (212.0, 386.9)	23	153.0 (90.5, 215.6)	0.50 (0.30, 0.83)^**^	0.49 (0.29, 0.83)^**^
**Age**, **≥75**^c^						
Influenza season	20	189.9 (106.6, 273.1)	17	155.1 (81.3, 228.8)	0.79 (0.41, 1.51)	0.82 (0.42, 1.59)
Non-influenza season	9	85.4 (29.6, 141.3)	19	173.3 (95.4, 251.2)	1.87 (0.84, 4.17)	2.06 (0.91, 4.64)
All season	29	275.3 (175.1, 375.5)	36	328.3 (221.1, 435.6)	1.13 (0.69, 1.84)	1.20 (0.73, 1.98)
**Female** ^d^						
Influenza season	27	148.8 (92.7, 204.9)	19	108.7 (59.8, 157.6)	0.71 (0.40, 1.29)	0.79 (0.43, 1.43)
Non-influenza season	17	93.7 (49.1, 138.2)	16	91.5 (46.7, 136.4)	0.87 (0.44, 1.74)	0.91 (0.45, 1.84)
All season	44	242.5 (170.8, 314.1)	35	200.2 (133.9, 266.5)	0.78 (0.50, 1.22)	0.84 (0.53, 1.32)
**Male** ^e^						
Influenza season	33	145.7 (96.0, 195.4)	16	72.9 (37.2, 108.6)	0.49 (0.27, 0.89)^*^	0.48 (0.26, 0.88)^*^
Non-influenza season	29	128.0 (81.4, 174.6)	18	82.0 (44.1, 119.9)	0.62 (0.34, 1.11)	0.55 (0.30, 1.01)
All season	62	273.7 (205.6, 341.9)	44	200.4 (141.2, 259.7)	0.55 (0.36, 0.83)^**^	0.52 (0.34, 0.79)^**^

a *Total follow-up 15234.5 person-year for unvaccinated and 13436.7 for Vaccinated*.

b *Total follow-up 15027.5 person-year for unvaccinated and 15031.2 for Vaccinated*.

c *Total follow-up 10534.5 person-year for unvaccinated and 10964.2 for Vaccinated*.

d *Total follow-up 18146.1 person-year for unvaccinated and 17481.2 for Vaccinated*.

e *Total follow-up 22650.3 person-year for unvaccinated and 21950.9 for Vaccinated*.

* *p < 0.05;*

** *p < 0.01*.

*C.I., confidence interval*.

*HR, hazard ratio*.

† *Main model is adjusted for COPD-related inpatient visits, COPD medications, total COPD medications, age, gender, CHA2DS2-VASc score, asthma, heart failure, acute myocardial infarction, stroke, ischemic heart disease, angina, peripheral vascular disease, hypertension, diabetes, depression, renal failure, chronic liver disease, dementia, level of urbanization, monthly income, antiarrhythmic agents: class1 (sodium channel blocker), class2 (beta blocker), class3 (potassium channel blocker), class4 (calcium channel blocker), aspirin, statin, RAASI*.

Male patients with COPD had a low occurrence of VA after receiving influenza vaccination in all seasons and influenza season (aHR: 0.52 [95% CI: 0.34–0.79] and 0.48 [95%CI: 0.26–0.88], respectively). Regarding female patients with COPD, no significant reduction in VA occurrence was observed between the vaccinated and unvaccinated groups in all seasons, influenza season, and non-influenza season.

### Association Between the Total Number of Influenza Vaccinations and VA

In the main model, there was no significant difference in VA occurrence after receiving one time, two to three, and more than four times influenza vaccination during the influenza season (Table 3). In the subgroup analysis, significant lower risk of VA occurrence was observed among patients without heart failure, ischemic heart disease, angina, peripheral vascular disease, or renal failure after receiving one time of vaccination in influenza season (aHR: 0.37 [95% CI: 0.15–0.94], 0.33 [95% CI: 0.12–0.93], 0.44 [95% CI: 0.20–0.98], 0.46 [95% CI: 0.22–0.97], respectively).

**Table 3:** Sensitivity analysis of adjusted HRs of vaccination in risk reduction of ventricular arrhythmia in influenza season.

	**Unvaccinated**	**Vaccinated**		
		**1**	**2–3**	**≥4**
	**Adjusted HR (95% C.I.)**	**Adjusted HR (95% C.I.)**	**Adjusted HR (95% C.I.)**	**Adjusted HR (95% C.I.)**
**Unadjusted**	1.00	0.53 (0.27, 1.05)	0.73 (0.42, 1.26)	0.47 (0.22, 0.98)^*^
**Model1^†^**	1.00	0.50 (0.25, 0.98)^*^	0.71 (0.41, 1.24)	0.47 (0.22, 0.99)^*^
Main model^‡^	1.00	0.52 (0.27, 1.03)	0.77 (0.44, 1.33)	0.54 (0.25, 1.15)
**Subgroup effects**				
Age, years				
55–74	1.00	0.08 (0.01, 0.60)^*^	0.69 (0.34, 1.41)	0.73 (0.32, 1.67)
≥75	1.00	1.19 (0.54, 2.65)	0.87 (0.36, 2.09)	0.18 (0.02, 1.40)
Gender				
Female	1.00	0.55 (0.21, 1.44)	1.04 (0.49, 2.19)	0.75 (0.25, 2.21)
Male	1.00	0.51 (0.20, 1.33)	0.52 (0.22, 1.19)	0.39 (0.14, 1.13)
COPD-related inpatient visits				
0	1.00	0.46 (0.18, 1.18)	1.10 (0.57, 2.12)	0.68 (0.26, 1.77)
1	1.00	0.88 (0.24, 3.26)	0.23 (0.05, 1.07)	
≥2	1.00	0.59 (0.12, 2.83)	0.53 (0.11, 2.56)	0.95 (0.24, 3.82)
CHA_2_DS_2_-VASc score				
0	1.00	0.30 (0.07, 1.36)		0.57 (0.15, 2.16)
1	1.00			
2–3	1.00	0.32 (0.07, 1.39)	0.33 (0.09, 1.12)	0.60 (0.20, 1.84)
≥4	1.00	0.86 (0.38, 1.91)	1.27 (0.63, 2.56)	0.18 (0.02, 1.35)
Asthma				
No	1.00	0.80 (0.37, 1.77)	0.78 (0.35, 1.72)	0.63 (0.22, 1.81)
Yes	1.00	0.23 (0.05, 0.97)^*^	0.78 (0.36, 1.68)	0.50 (0.17, 1.46)
Heart failure				
No	1.00	0.37 (0.15, 0.94)^*^	0.72 (0.37, 1.37)	0.59 (0.26, 1.33)
Yes	1.00	0.88 (0.31, 2.45)	0.93 (0.32, 2.69)	0.30 (0.04, 2.44)
Stroke				
No	1.00	0.51 (0.21, 1.21)	0.56 (0.26, 1.21)	0.57 (0.23, 1.37)
Yes	1.00	0.54 (0.18, 1.59)	1.11 (0.49, 2.51)	0.43 (0.10, 1.89)
Ischemic heart disease				
No	1.00	0.33 (0.12, 0.93)^*^	0.58 (0.27, 1.27)	0.77 (0.34, 1.77)
Yes	1.00	0.78 (0.31, 1.96)	0.98 (0.44, 2.16)	0.18 (0.02, 1.40)
Angina				
No	1.00	0.44 (0.20, 0.98)^*^	0.73 (0.40, 1.34)	0.53 (0.24, 1.18)
Yes	1.00	0.78 (0.20, 3.10)	1.04 (0.25, 4.28)	0.72 (0.08, 6.35)
Peripheral vascular disease				
No	1.00	0.47 (0.22, 1.00)^*^	0.57 (0.30, 1.11)	0.61 (0.29, 1.32)
Yes	1.00	0.87 (0.17, 4.42)	1.38 (0.41, 4.68)	
Hypertension				
No	1.00	0.36 (0.13, 0.95)^*^		0.63 (0.18, 2.19)
Yes	1.00	0.80 (0.40, 1.63)	0.86 (0.44, 1.68)	0.53 (0.20, 1.38)
Diabetes				
No	1.00	0.52 (0.23, 1.16)	0.47 (0.22, 1.01)	0.62 (0.27, 1.42)
Yes	1.00	0.53 (0.15, 1.83)	1.51 (0.65, 3.51)	0.29 (0.04, 2.26)
Renal failure				
No	1.00	0.46 (0.22, 0.97)^*^	0.74 (0.41, 1.32)	0.50 (0.22, 1.12)
Yes	1.00	1.51 (0.26, 8.68)	1.72 (0.29, 10.08)	1.03 (0.09, 11.29)
Chronic liver disease				
No	1.00	0.48 (0.22, 1.08)	0.70 (0.37, 1.34)	0.69 (0.32, 1.49)
Yes	1.00	0.68 (0.19, 2.43)		0.72 (0.24, 2.13)

* *p < 0.05; HR, hazard ratio*.

† *Model1 is adjusted for COPD-related inpatient visits, age, gender, CHA2DS2-VASc score, asthma, heart failure, acute myocardial infarction, stroke, ischemic heart disease, angina, peripheral vascular disease, hypertension, diabetes, depression, renal failure, chronic liver disease, dementia, level of urbanization, monthly income*.

‡ *Main model is adjusted for COPD-related inpatient visits, COPD medications, total COPD medications, age, gender, CHA2DS2-VASc score, asthma, heart failure, acute myocardial infarction, stroke, ischemic heart disease, angina, peripheral vascular disease, hypertension, diabetes, depression, renal failure, chronic liver disease, dementia. level of urbanization, monthly income, antiarrhythmic agents: class1 (sodium channel blocker), class2 (beta blocker), class3 (potassium channel blocker), class4 (calcium channel blocker), aspirin, statin, RAASI*.

During non-influenza season, there was no significant difference in VA occurrence after receiving one, two to three, and more than four times of vaccination (Table 4). Among patients with age between 55 and 74 years, a significantly lower risk of VA occurrence was observed after receiving one and two to three times of vaccination (aHR: 0.17 [95% CI: 0.04–0.73], 0.27 [95% CI: 0.10–0.77], respectively).

**Table 4:** Sensitivity analysis of adjusted HRs of vaccination in risk reduction of ventricular arrhythmia in non-influenza season.

	**Unvaccinated**	**Vaccinated**		
		**1**	**2–3**	**≥4**
	**Adjusted HR (95% C.I.)**	**Adjusted HR (95% C.I.)**	**Adjusted HR (95% C.I.)**	**Adjusted HR (95% C.I.)**
**Unadjusted**	1.00	0.67 (0.35, 1.32)	0.61 (0.32, 1.19)	0.89 (0.47, 1.69)
**Model1^†^**	1.00	0.65 (0.33, 1.26)	0.59 (0.30, 1.15)	0.87 (0.46, 1.66)
Main model^‡^	1.00	0.65 (0.33, 1.26)	0.60 (0.31, 1.16)	0.86 (0.45, 1.65)
**Subgroup effects**				
Age, years				
55–74	1.00	0.17 (0.04, 0.73)^*^	0.27 (0.10, 0.77)^*^	0.69 (0.33, 1.47)
≥75	1.00	2.23 (0.87, 5.74)	2.09 (0.76, 5.76)	1.59 (0.41, 6.12)
Gender				
Female	1.00	0.81 (0.31, 2.08)	0.96 (0.37, 2.49)	1.04 (0.34, 3.22)
Male	1.00	0.50 (0.19, 1.33)	0.41 (0.16, 1.07)	0.77 (0.34, 1.72)
COPD-related inpatient visits				
0	1.00	0.50 (0.21, 1.22)	0.65 (0.30, 1.44)	0.67 (0.29, 1.56)
1	1.00	0.58 (0.09, 3.89)	0.30 (0.02, 4.19)	
≥2	1.00	1.29 (0.33, 5.04)	0.68 (0.14, 3.36)	2.47 (0.72, 8.45)
CHA_2_DS_2_-VASc score				
0	1.00	0.33 (0.04, 2.73)	0.66 (0.14, 3.26)	1.23 (0.29, 5.13)
1	1.00			
2–3	1.00	0.25 (0.06, 1.10)	0.20 (0.05, 0.87)^*^	0.80 (0.31, 2.06)
≥4	1.00	1.14 (0.48, 2.71)	1.08 (0.44, 2.69)	0.81 (0.23, 2.87)
Asthma				
No	1.00	0.97 (0.40, 2.37)	0.89 (0.35, 2.26)	1.07 (0.41, 2.78)
Yes	1.00	0.43 (0.15, 1.23)	0.43 (0.16, 1.13)	0.73 (0.29, 1.83)
Heart failure				
No	1.00	0.41 (0.16, 1.06)	0.62 (0.30, 1.30)	0.90 (0.45, 1.78)
Yes	1.00	1.62 (0.54, 4.90)	0.70 (0.14, 3.55)	0.59 (0.06, 5.45)
Stroke				
No	1.00	0.49 (0.19, 1.27)	0.56 (0.24, 1.28)	0.93 (0.43, 2.00)
Yes	1.00	0.94 (0.35, 2.50)	0.67 (0.22, 2.08)	0.75 (0.21, 2.73)
Ischemic heart disease				
No	1.00	0.73 (0.27, 1.96)	0.66 (0.25, 1.75)	1.43 (0.66, 3.09)
Yes	1.00	0.63 (0.25, 1.58)	0.60 (0.24, 1.50)	0.34 (0.08, 1.47)
Angina				
No	1.00	0.57 (0.26, 1.24)	0.52 (0.24, 1.11)	0.89 (0.45, 1.76)
Yes	1.00	1.05 (0.25, 4.40)	1.10 (0.25, 4.80)	0.72 (0.08, 6.85)
Peripheral vascular disease				
No	1.00	0.46 (0.21, 1.04)	0.48 (0.22, 1.03)	0.79 (0.39, 1.60)
Yes	1.00	2.49 (0.55, 11.25)	2.82 (0.55, 14.55)	1.60 (0.22, 11.48)
Hypertension				
No	1.00	0.62 (0.17, 2.24)	0.44 (0.10, 1.97)	1.67 (0.60, 4.66)
Yes	1.00	0.65 (0.30, 1.42)	0.64 (0.30, 1.35)	0.60 (0.25, 1.46)
Diabetes				
No	1.00	0.59 (0.24, 1.45)	0.35 (0.12, 1.01)	0.99 (0.46, 2.16)
Yes	1.00	0.67 (0.24, 1.84)	0.99 (0.40, 2.43)	0.64 (0.18, 2.25)
Renal failure				
No	1.00	0.52 (0.23, 1.17)	0.69 (0.34, 1.40)	0.93 (0.45, 1.90)
Yes	1.00	0.90 (0.24, 3.47)	0.17 (0.02, 1.44)	0.50 (0.09, 2.89)
Chronic liver disease				
No	1.00	0.73 (0.35, 1.53)	0.49 (0.22, 1.11)	0.78 (0.37, 1.65)
Yes	1.00	0.46 (0.10, 2.16)	1.07 (0.32, 3.57)	1.54 (0.39, 6.08)

* *p < 0.05; HR, hazard ratio*.

† *Model1 is adjusted for COPD-related inpatient visits, age, gender, CHA2DS2-VASc score, asthma, heart failure, acute myocardial infartion, stroke, ischemic heart disease, angina, peripheral vascular disease, hypertension, diabetes, depression, renal failure, chronic liver disease, dementia, level of urbanization, monthly income*.

‡ *Main model is adjusted for COPD-related inpatient visits, COPD medications, total COPD medications, age, gender, CHA2DS2-VASc score, asthma, heart failure, acute myocardial infarction, stroke, ischemic heart disease, angina, peripheral vascular disease, hypertension, diabetes, depression, renal failure, chronic liver disease, dementia, level of urbanization, monthly income, antiarrhythmic agents: class1 (sodium channel blocker), class2 (beta blocker), class3 (potassium channel blocker), class4 (calcium channel blocker), aspirin, statin, RAASI*.

During all seasons, a significant lower risk of VA occurrence was observed after receiving one time of vaccination in the main model (aHR: 0.58 [95% CI: 0.36–0.93]) (Table 5). There was no significant difference in VA occurrence after receiving two to three times and more than four times of vaccination. Among patients with age between 55 and 74 years, a significantly lower risk of VA was observed after receiving one and two to three times of vaccination (aHR: 0.13 [95% CI: 0.04–0.40], 0.48 [95% CI: 0.27–0.85], respectively). In patients without stroke, peripheral vascular disease, diabetes, and with CHA_2_DS_2_-VASc scores of 2–3, a significantly lower risk of VA occurrence was observed after receiving one time and two to three times of vaccination. In patients with a history of asthma, a significantly lower risk of VA occurrence was observed after the first time of vaccination (aHR: 0.33 [95% CI: 0.14–0.78]). In patients without heart failure, ischemic heart disease, hypertension, angina, and renal failure, significant lower risk of VA occurrence was observed after first time of vaccination (aHR: 0.39 [95% CI: 0.20–0.76], 0.47 [95% CI: 0.23–0.96], 0.26 [95% CI: 0.08–0.86], 0.50 [95% CI: 0.29–0.88], 0.48 [95% CI: 0.28–0.84]).

**Table 5:** Sensitivity analysis of adjusted HRs of vaccination in risk reduction of ventricular arrhythmia in all season.

	**Unvaccinated**	**Vaccinated**		
		**1**	**2–3**	**≥4**
	**Adjusted HR (95% C.I.)**	**Adjusted HR (95% C.I.)**	**Adjusted HR (95% C.I.)**	**Adjusted HR (95% C.I.)**
**Unadjusted**	1.00	0.60 (0.37, 0.96)^*^	0.68 (0.44, 1.03)	0.65 (0.40, 1.06)
**Model1^†^**	1.00	0.57 (0.35, 0.91)^*^	0.66 (0.43, 1.01)	0.65 (0.40, 1.05)
Main model^‡^	1.00	0.58 (0.36, 0.93)^*^	0.69 (0.45, 1.05)	0.70 (0.43, 1.14)
**Subgroup effects**				
Age, years				
55–74	1.00	0.13 (0.04, 0.40)^***^	0.48 (0.27, 0.85)^*^	0.72 (0.41, 1.25)
≥75	1.00	1.52 (0.83, 2.76)	1.22 (0.64, 2.35)	0.56 (0.19, 1.63)
Gender				
Female	1.00	0.66 (0.34, 1.29)	1.02 (0.57, 1.84)	0.87 (0.40, 1.89)
Male	1.00	0.51 (0.26, 1.01)	0.46 (0.25, 0.87)^*^	0.59 (0.31, 1.12)
COPD-related inpatient visits				
0	1.00	0.48 (0.25, 0.92)^*^	0.87 (0.53, 1.44)	0.69 (0.37, 1.30)
1	1.00	0.85 (0.30, 2.37)	0.23 (0.06, 0.79)^*^	
≥2	1.00	0.87 (0.32, 2.38)	0.56 (0.19, 1.69)	1.65 (0.69, 3.95)
CHA_2_DS_2_-VASc score				
0	1.00	0.15 (0.02, 1.11)	0.57 (0.19, 1.69)	1.12 (0.44, 2.88)
1	1.00			
2–3	1.00	0.28 (0.10, 0.80)^*^	0.27 (0.10, 0.68)^**^	0.71 (0.35, 1.46)
≥4	1.00	0.97 (0.54, 1.74)	1.19 (0.68, 2.07)	0.43 (0.15, 1.21)
Asthma				
No	1.00	0.86 (0.48, 1.54)	0.81 (0.45, 1.49)	0.83 (0.41, 1.66)
Yes	1.00	0.33 (0.14, 0.78)^*^	0.60 (0.33, 1.10)	0.62 (0.31, 1.23)
Heart failure				
No	1.00	0.39 (0.20, 0.76)^**^	0.68 (0.41, 1.10)	0.75 (0.44, 1.26)
Yes	1.00	1.13 (0.54, 2.36)	0.86 (0.36, 2.05)	0.43 (0.10, 1.90)
Stroke				
No	1.00	0.50 (0.26, 0.95)^*^	0.55 (0.31, 0.97)^*^	0.74 (0.42, 1.31)
Yes	1.00	0.72 (0.35, 1.46)	0.93 (0.48, 1.79)	0.59 (0.23, 1.55)
Ischemic heart disease				
No	1.00	0.47 (0.23, 0.96)^*^	0.61 (0.33, 1.11)	1.03 (0.59, 1.79)
Yes	1.00	0.69 (0.36, 1.31)	0.78 (0.43, 1.42)	0.26 (0.08, 0.86)^*^
Angina				
No	1.00	0.50 (0.29, 0.88)^*^	0.64 (0.40, 1.02)	0.70 (0.42, 1.18)
Yes	1.00	0.84 (0.32, 2.21)	0.95 (0.35, 2.56)	0.66 (0.14, 3.08)
Peripheral vascular disease				
No	1.00	0.47 (0.27, 0.81)^**^	0.53 (0.32, 0.87)^*^	0.70 (0.42, 1.18)
Yes	1.00	1.41 (0.50, 3.94)	2.12 (0.83, 5.41)	0.53 (0.11, 2.59)
Hypertension				
No	1.00	0.26 (0.08, 0.86)^*^	0.57 (0.25, 1.30)	1.03 (0.48, 2.21)
Yes	1.00	0.73 (0.43, 1.23)	0.74 (0.45, 1.22)	0.57 (0.30, 1.09)
Diabetes				
No	1.00	0.55 (0.30, 1.00)^*^	0.43 (0.23, 0.79)^**^	0.79 (0.45, 1.39)
Yes	1.00	0.61 (0.28, 1.33)	1.21 (0.66, 2.23)	0.48 (0.17, 1.37)
Renal failure				
No	1.00	0.48 (0.28, 0.84)^**^	0.72 (0.46, 1.13)	0.69 (0.40, 1.17)
Yes	1.00	1.11 (0.41, 3.02)	0.49 (0.14, 1.77)	0.80 (0.21, 3.02)
Chronic liver disease				
No	1.00	0.60 (0.35, 1.03)	0.60 (0.36, 1.00)^*^	0.74 (0.43, 1.27)
Yes	1.00	0.54 (0.20, 1.45)	1.05 (0.48, 2.31)	0.59 (0.17, 2.04)

* *p < 0.05;*

** *p < 0.01;*

*** *p < 0.001; HR, hazard ratio*.

† *Model1 is adjusted for COPD-related inpatient visits, age, gender, CHA2DS2-VASc score, asthma, heart failure, acute myocardial infarction, stroke, ischemic heart disease, angina, peripheral vascular disease, hypertension, diabetes, depression, renal failure, chronic liver disease, dementia, level of urbanization, monthly income*.

‡ *Main model is adjusted for COPD-related inpatient visits, COPD medications, total COPD medications, age, gender, CHA2DS2-VASc score, asthma, heart failure, acute myocardial infarction, stroke, ischemic heart disease, angina, peripheral vascular disease, hypertension, diabetes, depression, renal failure, chronic liver disease, dementia, level of urbanization, monthly income, antiarrhythmic agents: class1 (sodium channel blocker), class2 (beta blocker), class3 (potassium channel blocker), class4 (calcium channel blocker), aspirin, statin, RAASI*.

During influenza season, non-influenza season, and all seasons, with more than two times of COPD-related inpatient visits, there was no significant difference of risk of VA occurrence after receiving one, two to three, or more than four times of vaccination. During all seasons, a lower risk of VA occurrence among patients without COPD-related inpatients after the first time of vaccination was observed (aHR: 0.48 [95% CI: 0.25–0.92]). In addition, among patients with one time of COPD-related admission, a lower risk of VA occurrence was associated with more than two times of vaccination (aHR: 0.23 [95% CI: 0.06–0.79]).

## Discussion

### Major Findings

In this population-based longitudinal cohort study that included 18,658 patients in Taiwan, the following principal findings were revealed: (1) influenza vaccination was associated with a significantly lower risk of potential lethal VA occurrence in patients with COPD, (2) more than one time of vaccination was associated with lower risk of VA occurrence, especially in patients with relative higher CHA_2_DS_2_-VASc score, and (3) lower risk of VA occurrence after the first time of vaccination was observed among patients without a history of stroke, ischemic heart disease, peripheral vascular disease, diabetes, and heart failure. After receiving two to three times of vaccination, a lower risk of VA occurrence was observed among patients without these medical histories except heart failure and ischemic heart disease.

To our knowledge, this is the first study to investigate the association between influenza vaccination and the risk of VA in patients with COPD.

### Mechanism of VA Development in Patients With COPD

A strong association between COPD and cardiovascular disease has been reported earlier, as they share similar risk factors (2). However, the definite mechanism linking VA and COPD is unclear despite earlier studies demonstrating COPD as an independent risk factor of sudden cardiac death (8). Konecny et al. reported that patients with COPD displayed a high risk of ventricular tachycardia regardless of left ventricular systolic function (6). Moreover, an increasing number of reports have revealed that the severity of COPD could be the leading cause of an increasing burden of VA (21, 22). Moreover, a prolonged QT interval in patients with COPD has been proposed to be a potential contributor to VA development (23). Other possible clinical manifestations, such as hypoxemia (24), pulmonary hypertension, and right ventricular failure, are also possible causes of the increased risk of VA among patients with COPD (25). Overall, patients with COPD are more prone to developing VA compared with healthy ones.

### Possible Mechanism of the Association Between Risk of VA and Influenza Vaccination

Earlier studies have revealed that influenza vaccination decreases not only the risk of developing atrial fibrillation (26) but also the risk of primary cardiac arrest in patients without a history of cardiovascular disease (14). In the present study, the risk of developing VA decreased significantly among patients with COPD who underwent vaccination. Among the patients with a concurrent diagnosis of asthma, which indicated the possibility of complex airway and lung parenchymal condition (27), also benefit from influenza vaccination.

A leading cause of VA is an acute coronary syndrome. In a study by Sung et al. (16) influenza vaccination decreases the risk of an acute coronary syndrome admission among elderly patients with COPD. Inflammation plays a critical role in the development of atherosclerotic plaque, thereby triggering acute coronary syndrome (28). During a viral infection of acute influenza, the increased metabolic demand, inflammatory conditions, platelet activation, and procoagulant status increase the risk of acute thrombus formation, subsequently developing into an acute coronary syndrome. Therefore, patients with COPD and ischemic heart disease might become free from VA development by preventing influenza infection upon undergoing vaccination. However, the present study showed that patients without risk of developing VA, such as patients without a medical history of hypertension, heart failure, renal failure, and ischemic heart disease, were associated with a lower risk of VA occurrence after receiving influenza vaccination.

Another possible cause of VA is inflammation of the myocardium caused by influenza infection (29). The incidence of myocarditis after influenza infection could be up to 9% according to a related study (30). Therefore, the risks of both myocarditis and VA occurrence are lowered upon vaccination as it prevents influenza infection.

Future research should focus on the mechanism through which the vaccine protects against VA development in patients with COPD to validate our results.

### Different Risks of VA Occurrence on Gender, Age, Comorbidities, and Influenza/Non-Influenza Seasons

Earlier studies have revealed that patients with a mean age of ~65 years had a low risk of major cardiovascular events upon receiving influenza vaccination (15, 31, 32). Our study provided more information; we observed that patients with COPD aged 65–74 years exhibited the greatest benefit from vaccination during influenza season, non-influenza season, and all seasons. In elderly patients (≥75 years), the risk of VA occurrence after the influenza vaccine did not significantly decrease. The elderly patients with COPD and ventricular arrhythmia had a high prevalence of multiple risk factors such as hypertension, coronary artery disease, and diabetes (6). The increased risk of VA development was possible because of increasing comorbidities and fragile health in these patients. In addition, the effectiveness of vaccination might be lowered in elderly patients because of lower immune response to influenza vaccination compared with younger patients (33, 34). In the present study, lower risk of VA occurrence was associated with influenza vaccination in the relative elderly COPD patients (55–74 years) after receiving more than one time of vaccination. Therefore, consecutive vaccination might be important among elderly COPD patients.

In patients with heart failure, peripheral vascular disease, renal failure, and chronic liver disease, the association was not as significant as patients without the above-mentioned comorbidities. The possible explanation is that the mechanisms of VA occurrence are complicated, and the effectiveness of influenza may be diminished while the presence of these comorbidities. However, in previous studies, the overall benefit from influenza vaccination to the patients with these diseases had been demonstrated (35–37). Therefore, annual influenza vaccination was recommended for these patients (38).

In a previous report from McLean et al. (39) the protective effects of the influenza vaccine against different types of viruses may be less notable following repeated vaccinations. However, annual vaccination is still recommended for the prevention and control of influenza infections (38). The fact that vaccine effectiveness in the United States during the 2017–2018 influenza season was only 38% (40), receiving vaccination only once may not be sufficient to prevent contracting the virus. With more than one vaccination, the risk of influenza infection may also decrease, as may the risk of further cardiovascular complications (16, 41), including ventricular arrhythmia.

In Taiwan, the influenza season is between October and March. During this period of significantly lower temperatures, an increased incidence of the acute coronary syndrome was observed (42). Increasing risk of ventricular arrhythmia during wintertime had been reported before (43). Moreover, increasing implantable cardiac defibrillator therapies was also observed during influenza season (44). However, the risk of myocardial infarction could increase even in hot weather (45). The present study demonstrated a significantly decreased risk of VA in the influenza season and all seasons among vaccinated patients, which echoes the results of an earlier study that the cardioprotective effects of the influenza vaccination were sustained beyond influenza season (4). A recent study by Kurihara et al. (46) revealed different pathogenesis of acute coronary syndrome in different temperatures. The predominant cause of acute coronary syndrome during seasons with relatively cold temperatures is plaque rupture, whereas plaque erosion is the major pathology in seasons with hot temperatures (46). Bradykinin 2 receptor-associated pathway and anti-inflammatory response were proposed as the potential mechanisms of the cardioprotective effects of the influenza vaccination (47, 48). Therefore, the decreasing risk of VA among vaccinated patients with COPD might be a consequence of plaque stabilization through all seasons.

An earlier study has reported the difference in the effects of the vaccination by gender in patients with COPD (49). In summary, female patients with COPD displayed more frequent respiratory symptoms, a higher prevalence of anxiety and depression, and better long-term outcomes compared with male patients with COPD (49). An earlier study demonstrated that female patients with COPD had a lower prevalence of ischemic heart disease but a significantly higher prevalence of chronic heart failure than male patients with COPD (50). In the present study, the risk of VA was not significantly different between vaccinated and unvaccinated female patients with COPD after propensity score matching for sociodemographic characteristics, comorbidities, and COPD medications. Future prospective studies that focus on gender differences in the protective effects of influenza vaccination against VA occurrence are necessary for validating this result.

### Impact of CHA_2_DS_2_-VASc Score and Hospitalization

We also observed that patients with COPD having a CHA_2_DS_2_-VASc score of 2–3 exhibited a low risk of VA occurrence after receiving vaccinations one and two to three times. An earlier study revealed that patients with atrial fibrillation having a high CHA_2_DS_2_-VASc score displayed a high risk of sudden cardiac death (51). This is because most of the components of the CHA_2_DS_2_-VASc score are also associated with an increased risk of VA occurrence. In the present study, we provided more information that could possibly predict the future benefit of vaccination among patients with COPD.

Another finding is that with fewer hospitalizations because of COPD, more benefits could be obtained from influenza vaccination. This result demonstrates that patients with poor pulmonary function or poor response to respiratory medications may not have long-term outcomes despite receiving the influenza vaccination. Earlier studies have demonstrated that influenza vaccination decreased the rate of acute exacerbation and hospitalization but did not reduce mortality rates (52, 53). During the exacerbation of COPD, the incidence of ventricular tachycardia was up to 25% (9). Moreover, acute right heart failure occur during exacerbation (54). The effectiveness of influenza vaccination might be diminished during an acute exacerbation. Another finding is that during the non-influenza season, only patients without any COPD-related inpatient visit had a lower risk of VA occurrence after receiving more than four times vaccination. A possible explanation is that during seasons with higher temperatures, the proportion of severe exacerbation is significantly higher than seasons with lower temperatures (55). Therefore, the importance of maintaining adequate pulmonary function to avoid exacerbation should be emphasized in patients with COPD.

### Limitations

The present study had several limitations. First, this is a retrospective observational study. Therefore, the presence of immortal time bias in observational studies could lead to the overestimation of the results (56, 57). In the present study, time-dependent Cox model analysis was performed to minimize immortal time bias (20). However, more future prospective studies are required to validate the findings of the present study. Second, the severity and diagnostic accuracy of COPD according to pulmonary function tests could not be obtained because of the lack of information from ICD-9 coding. However, we demonstrated that patients with few COPD hospitalizations benefit more from vaccinations compared with those with frequent hospitalizations. The readmission rate in patients with COPD is a marker of poor prognosis and is correlated with poor pulmonary function (58). Third, laboratory data, information of body mass index, and smoking status could not be collected because of information limitations from the NHIRD. Therefore, conducting future studies that link these patients to another registry is a possible solution for analyzing these possible cofounders. Fourth, the present study enrolled COPD patients with age more than 55 years because of the policy of free influenza vaccination. A future study is needed to investigate the younger COPD patients. Fifth, the information of rare hereditary or acquired diseases such as congenital heart defect, hypertrophic cardiomyopathy, long QT syndrome, or Brugada syndrome could not be collected from NHIRD. Future studies regarding the effectiveness of vaccination among COPD patients with these diseases should be investigated. Sixth, because of usually short-term prescription, the present study did not analyze the association between antibiotics and VA among patients with COPD. Seventh, the present study did not further evaluate the relationship between ventricular arrhythmia and sudden death. The cause of sudden death among patients with COPD is complicated and may be contributed by different conditions, such as arrhythmia, severe hypoxemia, respiratory failure, heart failure, sepsis, or ischemic heart disease (2, 59). Future study aimed to investigate the mechanism of sudden death in patients with COPD is warranted.

## Conclusion

The influenza vaccination may be associated with a lower risk of potential lethal VA occurrence in patients with COPD aged 55–74. Further future research is warranted to resolve this clinical question.

## Data Availability Statement

The data analyzed in this study is subject to the following licenses/restrictions: the use of the National Health Insurance Research Database needs to be applied for both IRB authority approval. The use of the database is also restricted to the approved entries. Requests to access these datasets should be directed to Ju-Chi Liu, liumdcv@tmu.edu.

## Author Contributions

C-CChe, W-RH, K-HC, C-CChi, TY, Y-WW, and J-CL contributed to the study conception and design. C-HL, J-SY, Y-AF, C-CChe, C-CChi, TY, and Y-WW contributed to data acquisition, analysis, and interpretation. The manuscript was drafted by C-CChe and C-HL and critically revised by all other co-authors. All authors read and approved the final version of this manuscript.

## Funding

This work was financially supported by the Higher Education Sprout Project by the Ministry of Education (MOE) in Taiwan and Taipei Medical University-Shuang Ho Hospital (Grant No. 109TMU-SHH-21).

## Conflict of Interest

The authors declare that the research was conducted in the absence of any commercial or financial relationships that could be construed as a potential conflict of interest.

## Publisher's Note

All claims expressed in this article are solely those of the authors and do not necessarily represent those of their affiliated organizations, or those of the publisher, the editors and the reviewers. Any product that may be evaluated in this article, or claim that may be made by its manufacturer, is not guaranteed or endorsed by the publisher.
